# Efficient preparation of Arabidopsis pollen tubes for ultrastructural analysis using chemical and cryo-fixation

**DOI:** 10.1186/s12870-017-1136-x

**Published:** 2017-10-27

**Authors:** Tohnyui Ndinyanka Fabrice, Andres Kaech, Gery Barmettler, Christof Eichenberger, J. Paul Knox, Ueli Grossniklaus, Christoph Ringli

**Affiliations:** 1 Department of Plant and Microbial Biology & Zurich-Basel Plant Science Center, University of Zurich, 8008 Zurich, Switzerland; 2 Center for Microscopy and Image Analysis, University of Zurich, 8057 Zurich, Switzerland; 3 University of Leeds, Center for Plant Sciences, Leeds, LS2 9JT UK

**Keywords:** Arabidopsis, Callose, Cell wall, Electron microscopy, Immunolabeling, Pollen, Pollen tube, Stylar tissue, Transmitting tract, Ultrastructure

## Abstract

**Background:**

The pollen tube (PT) serves as a model system for investigating plant cell growth and morphogenesis. Ultrastructural studies are indispensable to complement data from physiological and genetic analyses, yet an effective method is lacking for PTs of the model plant *Arabidopsis thaliana*.

**Methods:**

Here, we present reliable approaches for ultrastructural studies of Arabidopsis PTs, as well as an efficient technique for immunogold detection of cell wall epitopes. Using different fixation and embedding strategies, we show the amount of PT ultrastructural details that can be obtained by the different methods.

**Results:**

Dozens of cross-sections can be obtained simultaneously by the approach, which facilitates and shortens the time for evaluation. In addition to in vitro-grown PTs, our study follows the route of PTs from germination, growth along the pistil, to the penetration of the dense stylar tissue, which requires considerable mechanical forces. To this end, PTs have different strategies from growing between cells but also between the protoplast and the cell wall and even within each other, where they share a partly common cell wall. The separation of PT cell walls in an outer and an inner layer reported for many plant species is less clear in Arabidopsis PTs, where these cell wall substructures are connected by a distinct transition zone.

**Conclusions:**

The major advancement of this method is the effective production of a large number of longitudinal and cross-sections that permits obtaining a detailed and representative picture of pollen tube structures in an unprecedented way. This is particularly important when comparing PTs of wild type and mutants to identify even subtle alterations in cytoarchitecture. Arabidopsis is an excellent plant for genetic manipulation, yet the PTs, several-times smaller compared to tobacco or lily, represent a technical challenge. This study reveals a method to overcome this problem and make Arabidopsis PTs more amenable to a combination of genetic and ultrastructural analyses.

**Electronic supplementary material:**

The online version of this article (10.1186/s12870-017-1136-x) contains supplementary material, which is available to authorized users.

## Background

The pollen tube (PT) is an excellent model system for studying and probing biological processes at the single-cell level in plants. The PT emerges as a protuberance from a mature pollen grain when the conditions favour germination. In vivo, PTs grow through the stigmatic papillae into the style and eventually into the transmitting tract (TT) transporting the sperm cells to the embryo sac to effect double fertilization. The ability of PTs to grow in vitro makes them accessible for real-time probing of different cellular functions, such as vesicle trafficking dynamics [1, 2], the effect of pharmaceutical agents and ions on cell physiology and cellular organisation [3–7], the interaction of the PT with the pistil [8], and modelling the mechanics of PT growth [9, 10]. These approaches often require ultrastructural details at both the cell wall and/or cytoplasmic levels. Due to tip-localized growth, physiological processes such as cell wall assembly, endo/exocytosis, and the arrangement of cytoskeletal elements are asymmetrical across the length of the PT [11]. Thus, the knowledge of the cyto-architecture of the PT with spatial resolution along the tube is crucial for understanding the fine-tuning of PT growth. Despite the vast array of genetic resources available in Arabidopsis, of which a significant amount is associated with PT functions, and the considerable data on PT biology from live cell imaging techniques, such experiments often fail to provide a comprehensive picture of the ultimate biological role different factors play. This is mostly due to the lack of corroborative ultrastructural information. In contrast to PTs of other organisms such as lily [12], only a small portion of publications dealing with PT biology report the ultrastructure of Arabidopsis PTs [13–15], which are very delicate specimens. There is a lack of a detailed step-by-step description of reliable protocols for preparing Arabidopsis PT for ultrastructural analysis. There is also the need for an account on the ultrastructural details and the number of interpretable cross-sections that can be obtained by different fixation and embedding strategies. This is in spite the obvious necessity of reliable and highly detailed ultrastructural information.

Different approaches to ultrastructural studies vary from sample preparation and preservation/fixation, dehydration, resin type, and visualization. Whichever approach is chosen; it is advised to be aware of the critical steps and take adequate precautions. The preparation of biological specimen for ultrastructural studies begins with fixation. Fixation aims to arrest cellular activities and maintain the specimen in as near a natural state as possible. Chemical fixation (ChF) uses different combinations of chemicals, such as glutaraldehyde, osmium tetroxide, or uranyl acetate which stabilize the fine ultrastructural details of cells [16] by combining with cellular components to halt cellular activities and stabilise proteins [16–18] and lipids [19]. Cryo-fixation in combination with freeze substitution (FS) is the fixation method par excellence and the alternative to ChF. It can eliminate many artefactual damages [20] and improve the preservation of protein antigenicity [21]. Cryo-fixation consists of rapidly freezing specimens so that cellular processes and components are instantly immobilized and preserved in their natural state with high time resolution [22, 23]. Several cryo-fixation techniques exist such as slam freezing, propane jet freezing, plunge freezing (PF), and high pressure freezing (HPF). Here, we chose to describe PF and HPF due to the relative ease of preparation and excellent ultrastructural details that are obtained. Fixed specimens are embedded in resin (e.g. epoxy or acrylic resin) for sectioning. Epon is a light-coloured aliphatic-based epoxy resin routinely used in transmission electron microscopy (TEM; [24]). The acrylic LR white resin is an alternative to epoxy resins with very low viscosity, which allows to readily penetrate even into dense tissue [25]. Thin (60–80 nm) sections can be immunolabelled with antibodies against antigens on the specimen before contrast staining (usually with uranyl acetate and lead citrate) for visualization in the TEM.

Here, we present a simple and reproducible protocol to study the ultrastructure of PTs and the option of choosing a cost/time effective and reliable approach based on the goals and the resources available for the study. ChF, PF/FS and HPF/FS fixation techniques, Epon or LR white embedding, and immunogold labelling techniques are described. An assessment of the ultrastructural details obtained with any combination of steps is presented. The approach here offers the following advantages: 1) fine ultrastructural details of different subcellular structures are preserved, 2) the ease of visualizing the ultrastructure of PTs from apex to shank in a large sample size, the ultrastructure of many (up to 100) PTs can be analysed concurrently, 3) reliable and efficient immunogold localization and study of specific cell wall antigens (even on chemically fixed-Epon ChF/Epon sections). Our analysis reveals that in the stylar tissue, PTs can grow in the intercellular space or between the plasma membrane and the cell wall of cells of the TT, reflecting the mechanical strength of PTs. Frequently, PTs grow within each other, partly sharing a common cell wall. Importantly, it is becoming apparent that the outer and inner cell wall layers typically reported for PTs are, in Arabidopsis, connected by a distinct transition zone. Together, this study will enable to advance ultrastructural studies of PT growth dynamics including the vast array of mutants affecting PT functions.

## Methods

### Plant material

For Arabidopsis PTs, wild-type *Arabidopsis thaliana* seeds (accession Col-0) were surface sterilized with 1% sodium hypochlorite, 0.03% TritonX-100, plated and stratified on ½ strength Murashige and Skoog medium (containing 0.6% phytagel, 2% sucrose) for 2–4 days at 4 °C, and transferred to growth chambers with 16 h light and 8 h dark cycles at 22 °C. After 8 days, seedlings were transferred into pots containing soil and grown in the same growth chamber until flowering (about 4 weeks after potting).

### Semi-in vivo Arabidopsis pollen germination

**Fig. 1:**
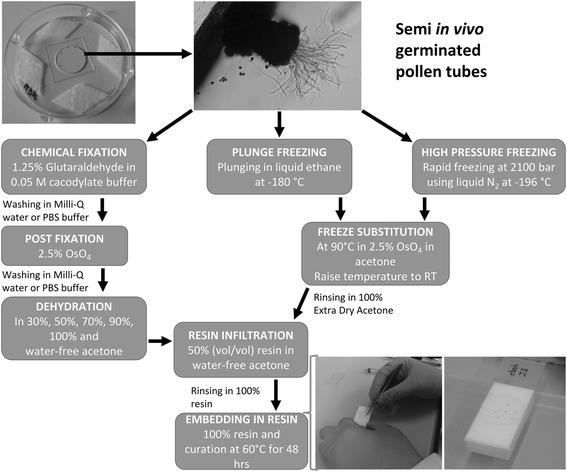
Schematic representation of sample preparation procedure by ChF, PF, and HPF from pollen germination to resin embedding. Arabidopsis PT preparation for ultrastructural studies showing the germination setup in the glass bottom petri dish, the germinated PTs that have grown through the stigma into the A-PGM, and the fixation, dehydration/freeze substitution, infiltration, and embedding steps. During embedding, individual specimens are carefully held with tweezers by the stigma of the pistil and slowly pulled along in the embedding resin to straighten out and align the PTs. A Teflon coated glass slide bearing a sample label is gently placed on top of the samples on the Teflon embedding mould (white block)

A list of materials required for all subsequent steps is provided as additional file 1. The sepals and petals of flowers that had opened on the same day were carefully removed and the stigmas were pollinated with pollen from the anthers. The pollinated pistils were cut just below the style (about 0.5 mm from stigmatic papillae) and transferred onto the Arabidopsis pollen germination medium (A-PGM) in a moist chamber glass bottom dish, ensuring that only the base (the ovary end) of the pistil was in contact with the A-PGM (Fig. 1). This set-up was incubated at 22 °C for 3.5 h, after which PTs had grown through into the A-PGM.

### Pollen tube fixation

#### Chemical fixation and dehydration

The samples were carefully picked up from the A-PGM by the stigmatic end and immediately transferred into the fixation solution. Samples were fixed at RT for 15 min and then on ice for 1 h 45 min, or at 4 °C overnight, then rinsed with Milli-Q water or PBS buffer 3 times for 10 min. This was followed by post-fixation with 1% osmium tetroxide on ice for 2 h and rinsing three times again as described. Serial dehydration of the samples was performed in 30% *v*/v, 50% v/v, 70% v/v, 90% v/v and 2 times in 100% acetone on ice for 10 min each, and the samples were moved into 100% water-free acetone for another 10 min, still on ice.

#### Cryo-fixation and freeze substitution

*High Pressure Freezing:* Arabidopsis samples were picked by the stigmatic end and placed on 10 μl A-PGM on 6 mm sapphire discs, while ensuring that the PTs did not curl around. Excess A-PGM was removed and drops of 1-hexadecene were added onto the specimen near the stigmatic end on the sapphire discs to ensure that the PTs remained longitudinally aligned. This set-up was completed with an aluminium specimen carrier and an additional 200 μm thick spacer ring of 6 mm diameter, and immediately frozen in an EM HPM100 high-pressure freezer (Leica Microsystems, Vienna, Austria). Frozen samples were transferred to and stored in liquid nitrogen until FS.

#### Plunge freezing

Samples were plunge frozen in liquid ethane using a custom-built, gravity-driven grid plunging device. Samples were placed on the 6 mm diameter sapphire disc (as described for HPF) held flat with a reverse tweezer dedicated to the grid plunger. The set-up was then mounted onto the arm of the grid plunger, excess medium was removed with filter paper, immediately plunge frozen in liquid ethane held at approximately −180 °C (just above the melting temperature), and stored in liquid nitrogen. Alternatively, 10 μl of 1-hexadecene (extracellular fluid) was added to the specimen (as describe for HPF) prior to freezing, but we observed no ultrastructural difference between PF specimen with and without 1-hexadecene.

#### Freeze substitution

Freeze substitution in water-free acetone with 1% OsO_4_ for 8 h at −90 °C, 7 h at −60 °C, 5 h at −30 °C, 1 h at 0 °C, with transition gradients of 30 °C per hour was performed using the Leica EM AFS 2. Samples were rinsed twice with water-free acetone, followed by embedding.

### Embedding

Samples were incubated in 1:1 Epon/water-free acetone or 1:1 LR white/water-free acetone at RT for 2 h and then transferred in 100% Epon embedding resin before embedding. Samples were infiltrated by serial steps with increasing resin concentrations in water-free ethanol: 33% *v*/v overnight at 4 °C, 66% v/v for 7 h at 4 °C, 100% LR white overnight at 4 °C. For embedding, samples were transferred into ~150 ml of degassed Epon embedding resin or 75 ml LR white on 100 μm deep Teflon embedding moulds. Specimens were gently held by the stigmatic end and dragged in the resin by a few millimetres to straighten out the PTs. Teflon-coated glass slides were then gently placed on the mould over the resin (Fig. 1) and this set-up was transferred into a 60–65 °C oven for 24–48 h for curing. LR white does not polymerise well in air, thus we ensured that the specimens were right in the middle of the resin before curing.

### Mounting, trimming and ultra-sectioning (TIMING 24 h)

**Fig. 2:**
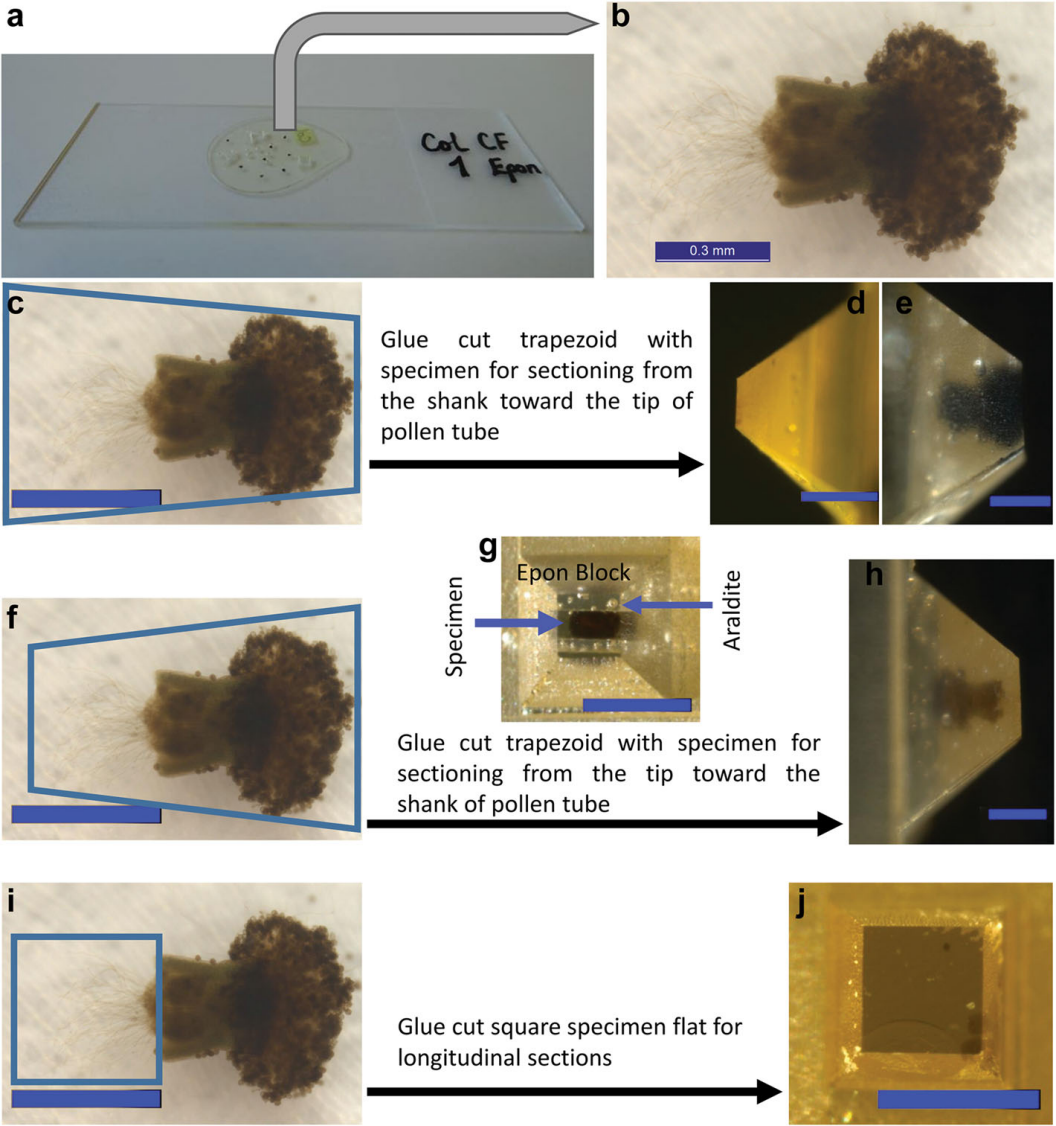
The embedded specimen and the three mounting and trimming modes for cutting transverse and longitudinal sectioning of PTs. **a** Cured resin sticks to the glass slide after separation from the mould. **b** Higher magnification of specimen showing the pistil and the PTs in the cured resin. **c** shows mode 1 for cutting and gluing sample onto resin block, and the corresponding block after trimming with the pistil removed (**d**) or left on (**e**). Mode 1 allows to cut transverse sections from the shank towards the tip of PTs. **f** shows mode 2 of cutting and gluing and the corresponding block after trimming in top view (**g**) and side view (**h**). In mode 2, transverse sections are cut from the tip towards the shank of PTs. In (**g**), hardened Araldite layer is distinguished from the specimen in the centre. **i** shows mode 3 of cutting and gluing samples onto the resin block and the corresponding trimmed block (**j**), allowing to cut longitudinal sections of PTs; the pistil is trimmed away. Scale bar: C–J = 500 μm

The polymerized specimens were plucked off (Fig. 2a), and an image was taken to measure the length of the PTs from the ovary end to the apex of the longest tubes (Fig. 2b). The samples were cut, glued with Araldite Standard onto polymerized Epon blocks, and let to harden overnight. Specimens were cut, glued, and trimmed for sectioning following three modes, which define whether transverse or longitudinal sections are produced (Fig. 2c-j). Trimming can be done with an electronic block trimmer or manually with a glass knife on the ultramicrotome (the method we used). Sectioning mode 1 (Fig. 2c-e): Transverse sections are produced from the shank towards the apex of the PTs. Trim the pistil away to section only PTs outside the TT, or leave parts of the pistil to visualize PTs within the pistil. Sectioning mode 2 (Fig. 2f–h): Transverse sections are produced from the apex towards the shank of the PTs. Care is required here to avoid having too much Epon/Araldite before the apex of the PTs, but also to avoid trimming the PTs away. Sectioning mode 3 (Fig. 2i–j): Longitudinal sections of PTs are produced on flatly glued specimen blocks. Flat embedded samples are about 100–150 μm thick and the PTs are concentrated in the second third of the specimen. Thus, trim away the first third (about 30–50 μm) of the block before taking semi-thin sections for toluidine blue staining. Then, cut semi-thin (200–350 nm thick) sections, stain with toluidine blue, and check under a bright field or phase contrast microscope for PTs (Additional file 1: Figure S1a, b). If no PTs are seen after toluidine blue staining, section ~10–20 μm into the block and repeat the staining until the PTs are seen. Then, cut and transfer ultra-thin (60–80 nm thick) sections onto the carbon-coated film surface on the slot grids, while recording the relative distance into the block.

### Immunogold labelling

In all the labelling and post-staining steps, drops of solutions are placed on Parafilm. Rehydrate grids with ultra-thin sections by floating the side with the sections face-down in drops of PBS blocking solution for 1 h. For immunogold labelling and subsequent staining of grids with uranyl acetate and lead citrate, take care to avoid breaking the Formvar/carbon coating as this will lead to loss of the sample. Move the grids onto a drop of fresh blocking solution containing an adequate dilution of the primary antibody (1:50 LM6, LM15, 1:150 Anti-callose), incubate for 2 h at RT or overnight at 4 °C. Rinse grids three times in drops of PBS buffer for 10 min to wash away any unbound or unspecifically bound antibodies. Make sure that grids do not dry out during any of the incubation steps as this may destroy the primary antibody and obstruct the binding of the secondary gold-coated antibody. Transfer grids onto drops of fresh blocking solution containing a 1:25 dilution of 10 nm gold-coated secondary antibody (anti-rat IgG (whole molecule)-gold antibody against LM6, LM15, and anti-mouse IgG (whole molecule)-gold antibody against anti-callose, and incubate for 1 h. Rinse grids 3 times in drops of PBS buffer and 2 times in Milli-Q water for 5 min per wash. The same resin-embedded sections can also be used for immunogold labelling of other pollen tube epitopes, as well as for light/fluorescence microscopy.

### Uranyl acetate and lead citrate staining

Stain grids by floating the section-side face-down on 40 μl drops of 2% uranyl acetate for 10 min. Rinse grids 4 times in drops of Milli-Q water, and dry them by carefully removing liquid with a filter paper. Some authors recommend drying grids for several hours after uranyl staining, but our approach of drying with filter paper and proceeding immediately to lead citrate staining produces good contrast. Stain the sections (Additional file 1: Figure S1c) in drops of lead citrate for 5 min. Rinse 4 times in drops of Milli-Q water and dry the grids by carefully removing the liquid with a filter paper, return grids into the grid box and dry at 37 °C for 15 min.

### TEM imaging

All thin sections were imaged in a CM 100 transmission electron microscope (FEI, Eindhoven, The Netherlands) at an acceleration voltage of 80 kV using a Gatan Orius 1000 CCD camera (Gatan, Munich, Germany). For immunogold-labelled sections, it can be difficult to distinguish cellular components like ribosomes, which may stain heavily with heavy metals and can look like gold particles. This can be resolved by varying the histogram on the live view mode of the camera to make clearer the difference in contrast. Membrane delimitations of membrane-bound structures can be difficult to resolve on one section only, due to the dependence on the angle at which the section cuts through the structures. By analysing consecutive serial sections, this problem can be easily resolved as some perspective on the 3D structure can be obtained. Using this approach, we could distinguish lipid droplets from vacuoles in HPF and PF sections.

## Results and discussion

### Ultrastructural details of transverse sections of PT depend on preparation method

For preparation of PTs for embedding, they were grown under semi-in vivo conditions, where stigmas are pollinated and cut just below the style (about 0.5 mm from stigmatic papillae), from where they grow through the stigmatic tissue into the growth medium. Semi-in vivo pollen germination offers several advantages and has been used to investigate genetic factors in pollen-pistil interactions [26], the dynamics of PT-pistil interactions, and, particularly, PT-ovule interactions [27–29]. PTs grown in this way are thought to be comparable to in vivo grown PTs. Arabidopsis stigmas were pollinated and transferred onto A-PGM in a moist chamber glass bottom dish, ensuring that only the base (the ovary end) of the pistil was in contact with the A-PGM. After growth of the PTs through the pistil into the A-PGM, the samples were fixed either by chemical fixation (ChF), Plunge Freezing (PF), or High Pressure Freezing (HPF), and embedded in Epon or LR white as outlined in Fig. 1 and described in a step-by-step protocol in the additional file 1. The preparation of embedded pistils/PTs was performed to allow the cutting of transverse sections from the tip or the base of the PTs, and of longitudinal sections as shown in Fig. 2.

**Fig. 3:**
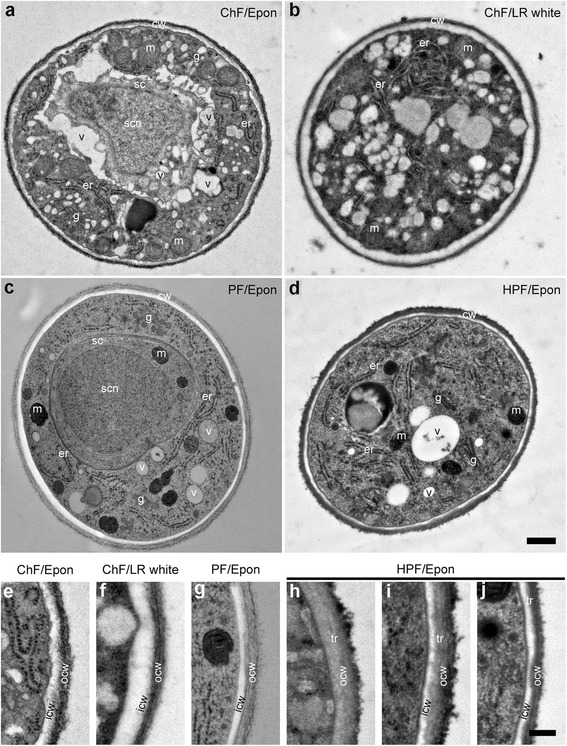
The transverse sections of ChF, PF and HPF PTs outside the pistil at similar distance from the tip showing the cell wall and cytoplasmic structures. **a**-**d** are entire transverse sections of PTs at about 40 μm from the apex showing the cell wall, and cytoplasmic features, including the sperm cell and sperm cell nucleus (**a**, **c**). The resolution of ChF/Epon section (**a**) is higher than ChF/LR white section (**b**). (**e**–**j**) are close-up views showing the cell wall layers: the inner (whitish/light grey) and outer (dark striated and fluffy) layers. A distinguishable transition layer (dark grey region) is seen between the outer and the inner layer in the HPF sections (**h**–**j**). The development of the cell wall layers is presented from the apical region (**h**) through sub-distal (about 25–35 μm from apex: [**i**]) to the distal about 40 μm toward the shank (**j**). The HPF section (**h**) of the apical cell wall where the callose layer (secondary/inner wall) is absent reveals that the primary cell wall of Arabidopsis PTs has two layers: an outer dark fluffy/hairy and an inner grey layer. In the shank where the secondary callose layer is formed (**i** and **j**), the three different wall layers are clearly visible. The bilayer of the plasma membrane is visible only in HPF sections. m = mitochondria, g = Golgi apparatus, er = endoplasmic reticulum, cw = cell wall, p = plasma membrane, icw = inner cell wall, ocw = outer cell wall, v = vacuole, sc = sperm cell, scn = sperm cell nucleus, tr = transition layer. Scale bar: (**a**–**d**) = 500 nm; (**e**–**j**) = 200 nm

First, the ultrastructure of Epon- and LR white-ChF embedded sections were compared. While Epon provides excellent preservation of morphological details, it co-polymerises with the specimen through the formation of hydrophobic crosslinks and covalent bonds with proteins, which may hinder antigen-antibody interactions. In contrast, LR white only self-polymerizes in the specimen, and thus offers little hindrance to antigen-antibody interactions [30, 31]. However, LR white can engender severe extraction of cellular constituents, resulting in poor ultrastructure [32, 33]. Consistent with these reported artefactual damages, higher ultrastructural resolutions and morphological details were obtained from Epon than from LR white sections (Fig. 3a, b). Some obvious ultrastructural artefacts from LR white included shrinkage at the plasma membrane/cell wall junction, low resolution of organelles, and reduced visibility of membranes.

Given the higher resolution, Epon was used to study the ultrastructural details after fixation with different methods (ChF, PF, and HPF) in transverse sections about 40 μm behind the apex of the PTs (Fig. 3a, c). In this region, the two cell wall layers were clearly distinguishable: an electron-weak inner and an electron-dense fibrillary outer layer. Earlier reports established that the PT cell wall is made of a primary pectin-rich outer wall and a secondary callose-rich inner wall [14, 15, 34]. Zooming in, we observed that the cell walls of PTs have a relatively electron transparent (whitish/light grey) inner layer and transitions through an electron-weak (greyish) layer to the electron-dense and fibrous (dark) outer layer, distinctions which were better resolved in HPF sections. This intermediate transition zone between the outer and inner cell walls is not observed in LR white- or spur resin-embedded material [14, 15]. The plasma membrane was best preserved and resolved in HPF/Epon (Fig. 3e–j). In ChF sections, some buckling of the plasma membrane was observed, while in PF and HPF sections, membranes appeared smooth and circular. Membrane artefacts that may be introduced by ChF were previously discussed [20, 30]. In some PF samples, while the boundary between the cytoplasm and the cell wall was clearly distinguishable, the plasma membrane appeared less resolved.

**Fig. 4:**
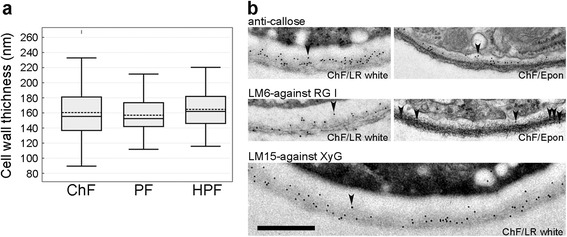
Cell wall thickness and immunogold localization of wall carbohydrate epitopes. **a** The mean and median values of PT cell wall thickness are shown by the dash-lines and the line in the middle of the box, respectively. The mean cell wall thickness is similar between ChF (160 nm, *n* = 100), PF (156 nm, *n* = 50), and HPF (164 nm, n = 100) sections. **b** Immunogold labelling (examples indicated by arrowheads) for callose (top panel), LM6 against (1–5)-α-L-arabinosyl residues of rhamnogalacturonan-I (RG-I, middle panel), and LM15 against the xyloglucan motif, (XXXG, bottom panel) of ChF/LR white and/or ChF/Epon sections. The labelling for callose localizes to the inner cell wall layer and serves as reference to distiguish the primary from the secondary cell wall. While the antigenicity is more highly preserved in ChF/LR white sections than ChF/Epon sections (as revealed by the relative higher abundance of the gold particles), there is considerably high shrinkage of the plasma membrane on LR white sections. The distributions of RG-I and xyloglucan occur in both the inner and outer cell wall layers. Scale bar = 500 nm

The outer wall appeared somewhat striated inwardly and fluffy/hairy outwardly (Fig. 3e-j). In about 50% of PF/Epon PTs, the outer wall layer almost entirely disappeared (additional file 2). The disappearance of the primary cell wall about 50 μm from the PT tip has previously been reported [13]. As this was only observed in PF-fixed tubes, but not in those fixed with either ChF or HPF, our results suggest that this apparent disappearance is likely an artefact of PF. Despite the subtle changes in ultrastructural appearances, the average thickness of the PT cell wall from the different fixation methods was similar at around 160 nm (Fig. 4a).

### Immunogold labelling allows detection of cell wall substructures and is more effective in LR white-embedded material

**Fig. 5:**
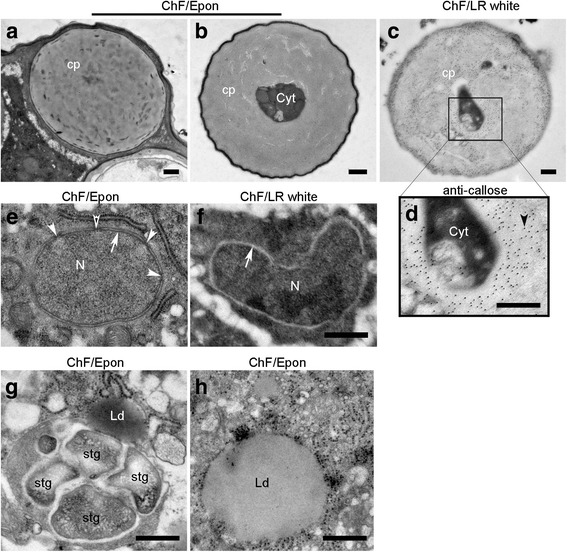
Detailed intracellular ultrastructural features of PTs showing callose plugs, the nucleus, starch grains, and lipid bodies. ChF section through callose plugs (cp) of a PT with completely sealed off cytoplasm (**a**) or a plug closing in on remnant of the cytoplasm (**b**). The ultrastructure is more preserved in the ChF/Epon sections (**a**, **b**) than the ChF/LR white section (**c**). The closer view of the area marked in the LR white section showing anti-callose gold particle labelling indicated by an arrowhead (**d**). Sections through the nucleus (N) in ChF/Epon (**e**) and ChF/LR white (**f**). While the nuclear membrane is well resolved showing its two layers (arrow) and nuclear pores (arrowheads) in ChF/Epon section, the membrane is entirely extracted in ChF/LR white section and appears electron transparent (arrow). ChF/Epon sections show starch grains (stg) in amyloplasts (**g**) and a lipid body (Ld) surrounded by ribosomes (**h**). Lipid bodies are only clearly preserved and contrasted on ChF/Epon section. Scale bar (**a**–**h**) = 500 nm

Immunogold labelling can be used to investigate the intracellular dynamics of some crucial molecular players in PT function, including cell wall components. Monoclonal antibodies targeting plasma membrane-synthesized [anti-(1–3)-β-glucan monoclonal antibody against callose] and Golgi-packaged [LM6 against (1–5)-α-L-arabinosyl residues of rhamnogalacturonan-I (RG-I), and LM15 against the xyloglucan motif (XXXG)] cell wall epitopes were used to investigate efficiency and distribution of immunogold signals. While the labelling efficiency in ChF/LR white sections was stronger than in ChF/Epon sections (Fig. 4b), the results were qualitatively the same for both embedding resins. Callose is absent from the outer wall and found only in the inner, electron-weaker wall, confirming previous findings [14, 15], and the transition zone between the inner and the outer cell wall layer. While LM15 labelling was consistent with reports by others, we found a broader distribution of the LM6 epitope in the outer and inner wall, rather than a restriction to the outer wall layer [14, 15]. Distal from the tip region, callose plugs are formed that partially or completely seal off the cytoplasm and are strongly and regularly labelled with the anti-callose antibody (Fig. 5a-d). While they are not essential for PT growth per se, callose plugs influence cell wall patterning and limit the viable cytoplasm of the PT to the region between the tip and the first plug [35–38].

### Intracellular ultrastructural details

Appreciable details of subcellular structures were obtained from ChF, PF, and HPF sections of PTs; features such as the sperm cells, vegetative nucleus of the PT, mitochondria, ribosomes, ER, Golgi, secretory vesicles, starch granules, lipid droplets, and vacuoles were seen. In ChF/Epon sections, intracellular vacuoles were slightly shrunken and distorted, leading to buckling of the membranes, but their profiles were smooth and circular in PF and HPF sections (Fig. 3a–d). Nuclear membranes appeared as two black lines in Epon-embedded material, whereas the lipid double-layer was less clearly visible in LR white (Fig. 5e, f). Starch granules were contained within structures like amyloplasts (Fig. 5g). In ChF/Epon sections, lipid droplets were also clearly distinguishable from vacuoles by their staining with heavy metals (Fig. 5h), while their content seemed to be extracted in PF and HPF sections as they appeared electron transparent. Lipid droplets were distinguished from vacuoles in PF and HPF sections as spherical structures with no internal structure and no resolvable membrane delimitation (additional file 3). In PF sections, mitochondria occasionally appeared so strongly contrasted that the cristae are largely obscured. A summary of the degree of ultrastructural preservation and resolution of cellular substructures by ChF, PF, and HPF is presented in Table 1.

**Table 1:** Summary of the degree of ultrastructural preservation and resolution of cellular substructures by ChF, PF and HPF

Cellular Substructure	Ultrastructure preservation and resolution			
	ChF		PF	HPF
	LR white	Epon	Epon	Epon
Cell Wall	2	4	3	5
Plasma membrane	1	3	4	5
Golgi complex	1	3	4	5
Mitochondria	2	4	3	4
Endoplasmic reticulum	3	5	5	5
Ribosomes	4	5	5	5
Vacuole	2	3	5	5
Vacuole integration	na	na	4	5
Vesicles	1	3	4	5
Lipid droplets	na	4	2	2
Starch granules	2	4	4	5
Microtubules	na	na	na	5
Sperm cells	3	4	4	5
Nucleus	3	4	4	5
Callose Plugs	4	5	5	5
Cytoplasmic organization	2	4	4	5
Ice crystals damages	na	na	3	1

The quality of the ultrastructure is rated on a 5 point bases as follows: 1) ultrastructural features unresolvable, the substructure is barely visible; 2) substructure is visible; 3) substructure and some ultrastructural features distinguishable; 4) efficient preservation and resolution of most details of the substructure; 5) fine preservation and resolution of substructure and clear distinction of ultrastructural details, and assumed to be the closest to the native structure. na = not applicable or substructure is not visible

### Longitudinal sections through Arabidopsis PTs

**Fig. 6:**
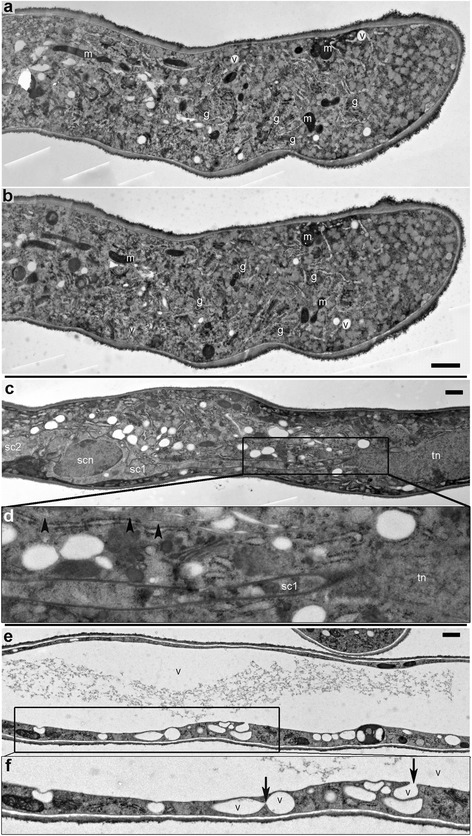
Longitudinal section of HPF PTs outside the pistil showing different regions of the cytoplasm. The cyto-architecture across the pollen tube apical region (**a**, **b**), the male germ unit region (**c**, **d**) and the large vacuolar region (**e**, **f**). (**a**) and (**b**) show two longitudinal sections of the same PT about 200–250 μm apart, providing a glimpse into the 3D ultrastructure. The cell wall in the apical region has different thicknesses across the length. An apical cytoplasmic-clear region within 4–5 μm of the apex is full of small vesicles; large organelles are only present distally. In (**c**) a sperm cell is distinguished from the tube nucleus by the inclusion of their nucleus within membrane delimited cytoplasm. **d** is a close-up view of the marked area in (**c**) showing a cytoplasmic extension of sperm cell I (sc1) connecting to the tube nucleus (tn); microtubule arrays (arrowheads) can also be seen in this region. In (**e**) and (**f**), the large central vacuole is shown. **f** is a close-up view of the marked area in (**e**) showing the sites of vacuole integration (arrows); equally the inner callose, the transition zone, and outer fibrous layers of the cell wall are visible. m = mitochondria, g = Golgi apparatus, er = endoplasmic reticulum, cw = cell wall, v = vacuole, scn = sperm cell nucleus, sc1 = sperm cell 1, sc2 = sperm cell 2, tn = tube nucleus. Scale bars = 1 μm

Since the ultrastructure was best preserved and resolved after HPF, longitudinal sections of HPF specimens were considered for describing the cyto-architecture of Arabidopsis PT (that is, the organization/distribution of intracellular features such as Golgi, ER, vacuoles, vesicles, microtubules, the nuclei, and PT cell wall) along its length (Fig. 6). We distinguished three regions along the length of the PT: the apical region (Fig. 6a, b), the male germ unit region (vegetative nucleus and the two sperm cells) (Fig. 6c, d), and the large vacuolar region (Fig. 6e, f). Fig. 6a and b show ultrastructural details from two sections of the same pollen tube about 210 nm apart. Note the variation in the amount of ultrastructural details of intracellular components between them. This is due to the orientation of the objects relative to the section. A series of cuts could be reconstituted by 3D tomography, which would give more insight into the three dimensions of subcellular structures. The apical cell wall showed two distinct cell wall formations (an inner grey and an outer fluffy dark layer: Fig. 3i; Fig. 6a, b) and a slightly variable thickness along the length of the PT, possibly reflecting oscillatory growth. The electron-weak callose layer (whitish/light grey) was not yet formed here. The apical cytoplasmic clear zone, which is full of small vesicles and contains no large organelles, was also visible within 4–5 μm from the apex.

In the male germ unit region, the two sperm cell nuclei are distinguished from the vegetative nucleus, which remains loose within the PT cytoplasm, by their enclosure within membrane-bound compartments (Fig. 6c). Sperm cell I (SC1) is seen stretching across to the tube nucleus by a cytoplasmic extension of about 10 μm (Fig. 6d). Arrays of longitudinally arranged microtubules are visible in this region (Fig. 6d).

In the vacuolar region, the sites of vacuole fusion are seen (Fig. 6e, f), resulting in the large central vacuole common to most plant cell types [39]. The genetic basis for the biogenesis of vacuoles and the role of vacuoles in regulating plant cell morphogenesis and homeostasis, particularly for PT growth, is critical [40–42].

### PTs in the pistil

**Fig. 7:**
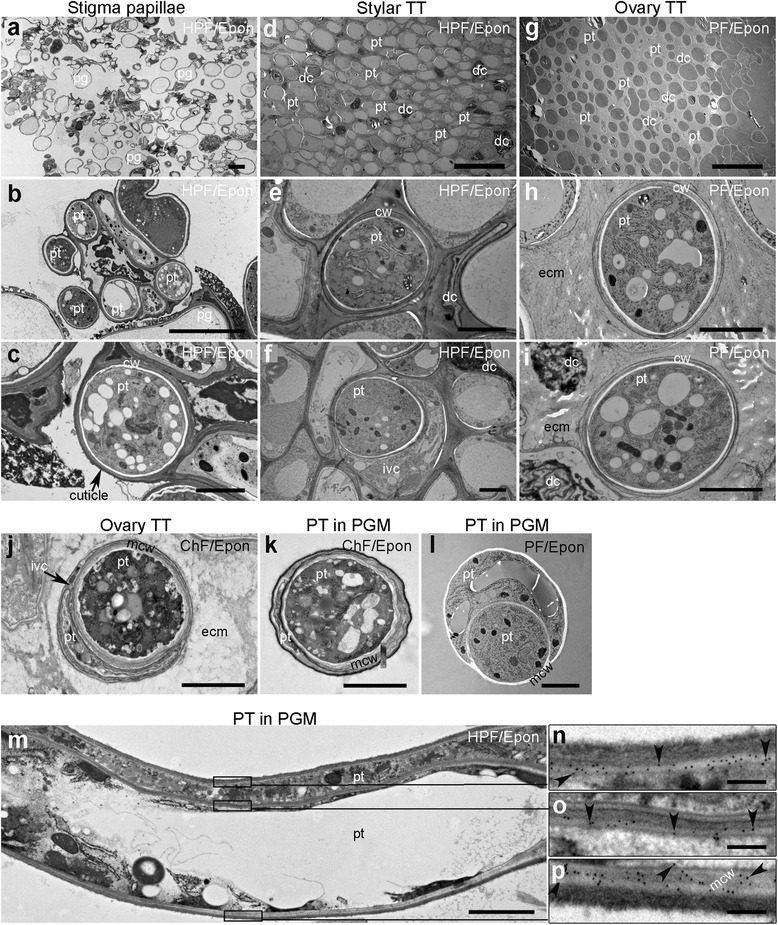
Section through the stigma, style, and ovary showing pollen tube growth/path through the transmitting tract of the pistil and the degree of ultrastructure preservation by HPF, PF, and ChF. The PT path through the stigma surface (**a**–**c**), the stylar TT (**d**–**f**), and the ovary TT (**g**–**i**). (**a**–**f**) HPF/Epon sections and (**g**–**i**) PF/Epon sections. **b** and **c** are increasing magnifications of (**a**) showing PTs that have grown through the apoplastic matrix of stigmatic papillar cells. **d** shows very densely packed cells in the style. Here, PTs need to overcome the mechanical resistance from the tightly packed surrounding cells in a fashion that requires more invasive growth potentials than elsewhere in the TT. Higher magnifications show PTs displacing TT cells and growing through the intercellular matrix (**e**) or the apoplastic space (**f**). (**g**) shows more loosely distributed cells within the ovary TT, where spent PT and degenerating TT cells are also visible. Higher magnifications show that cells are lodged in an extensive extracellular matrix, in which PTs can be seen to have cell walls with layers of different electron densities (**h** and **i**). (**j**) shows two PTs within a spent TT cell, one of the tubes is apparently degenerating while the other seems to have an active cytoplasm and fills up the larger volume. The extracellular matrix is clearly more preserved in the cryo-fixed section (**h** and **i**) than in the ChF section (**j**). (**k** and **l**) show transverse sections of in vitro-grown PTs contained within another with a region where their two cell walls are merged (mcw). The ultrastructure details presented of mcw appear different in the ChF (extensive fibrous, **k**), PF (almost electron transparent, **l**) and HPF (fibrous/dark gray, **m**) sections. **l** also reveals the artefactual loss of the primary (outer) cell wall layer in PF sections. Higher magnifications show anti-callose gold particle labelling (indicated by arrowheads) revealing callose distribution/extent in the unmerged cell wall of the outer PT (**n**), the unmerged cell wall of the inner PT (**o**), and the merged cell walls (**p**) corresponding to the marked regions in (**m**), respectively. pg = pollen grain, pt. = pollen tube, cw = cell wall, aps = apoplastic space, icm = intercellular matrix, ecm = extracellular matrix, dc = spent/degenerating cells, mcw = merged cell walls. Scale bar (**a**, **d**, **g**) = 20 μm; (**b**, **c**, **e**, **f**, **h**–**m**) = 2 μm; (**n**–**p**) = 200 nm

Finally, the structural details of PTs growing in the TT were investigated in more detail. By sectioning as shown in Fig. 2e, ultrastructural features of PTs within the pistil were less well resolved after ChF and, while fine ultrastructural details of PTs within the TT were resolvable in PF samples, finer details were preserved and resolved after HPF, which is the superior cryo-fixation technique compared to CHF in the preservation of specimens of a thickness up to hundreds of microns in a single shot [21, 43] (Fig. 7). After germination on the pistil (Fig. 7a-c), PTs grow along the pistil cells [44] through the tight stylar tissue in the intercellular space between cells, or between the cell wall and the protoplast (Fig. 7d-f), indicating a strong mechanical resistance that needs to be overcome by PTs in order to reach the TT. Interestingly, PTs were frequently found to grow within each other and even to share a common callose layer (Fig. 7j), as described earlier [38]. This phenomenon was also observed in PTs grown in vitro (Fig. 7k, l). Also in longitudinal sections, engulfment of PTs was observed (Fig. 7m), and callose deposition revealed by antibody labelling (Fig. 7n-p) confirmed 1 PT within another. Following the stylar tissue, cells of the transmitting tract are more loosely arranged, with extensive intercellular space where viable and spent PTs can be distinguished (Fig. 7g-i), the latter being the distal part of the PT, separated from the viable cytoplasm by callose plugs (Lord, 2003).

## Conclusions

The method described here allows the concomitant analysis of a large number of PTs for growth and development at different stages, along the PT axes, and in the various tissues of the TT as well as in vitro. Fixation and embedding strategies depend on the information one wishes to retrieve from the analysis and are also determined by the tissue to be analysed, i.e. whether PTs are grown in vitro or embedded in the female tissue. In essence, Epon embedding is the method of choice for ultrastructural studies whereas immunolabelling is more effective with LR white-embedded PTs. The procedure results in a large number of sections of defined orientation, a prerequisite for a quantitatively and qualitatively effective analysis that is essential for obtaining a representative picture of PT structure and growth, and particularly important when comparing, e.g., wild-type with mutant PTs showing subtle alterations in cyto-architecture. Despite differences in the quality of preservation of the subcellular structures, principal parameters such as PT diameter or cell wall thickness are comparable between samples fixed and embedded by the different strategies, demonstrating that comparison of these parameters between differently treated material is possible.

## Additional files

Additional file 1: Additional Methods. Detailed step-by-step procedure and list of material needed for growth of PTs and fixation, embedding, and sectioning of the specimen. (DOCX 56 kb)

Additional file 2: Loss of the primary cell wall in PF. PTs fixed by PF frequently showed loss of the primary (outer) cell wall layer, leaving the callose layer. This was not observed with the other fixation methods. Scale bar: 500 nm. (DOCX 250 kb)

Additional file 3: Comparison of the ChF, PF, and HPF ultrastructural details of PTs within the pistil. The intracellular ultrastructural details of PTs within the TT achieved by HPF (A), PF (B) and ChF (C). Except for the lipid droplets which are best resolved by ChF, the resolution of most intracellular features is highest in HPF, followed by PF and then ChF sections. Ld = Lipid bodies, g = Golgi, er = endoplasmic reticulum, v = vacuole, m = mitochondria Scale bar: 500 nm. (DOCX 531 kb)

## Abbreviations

A-PGM: Arabidopsis pollen germination medium
CHF: Chemical fixation
Col: Columbia (Arabidopsis accession)
FS: Freeze substitution
HPF: High pressure freezing
PF: Plunge freezing
PGM: Pollen germination medium
PT: Pollen tube
RG-I: Rhamnogalacturonan I
SC: Sperm cell
TEM: Transmission electron microscopy
TT: Transmitting tract
