# Supplementary material for: Efficient preparation of Arabidopsis pollen tubes for ultrastructural analysis using chemical and cryo-fixation
Source: BMC Plant Biol. 2017 Oct 27;17:176. doi: 10.1186/s12870-017-1136-x (PMC5658917; doi:10.1186/s12870-017-1136-x)
Supplement: Supplementary file 1 — Additional Methods. Detailed step-by-step procedure and list of material needed for growth of PTs and fixation, embedding, and sectioning of the specimen. (DOCX 56 kb) [file 12870_2017_1136_MOESM1_ESM.docx]

## Additional files

## Additional file 1

### Step-by-step protocol for fixation and embedding of samples

### Equipment and reagents (in approximate order of use)

Where CAUTION is indicated, the product is either irritating to eyes, respiratory system, and skin, toxic and/or potentially carcinogenic. Wear suitable gloves and avoid contact with skin and eyes. Dispose of the waste in appropriate containers. Check the material safety data sheet of each of the products for further details.

Procedure-specific reagent/equipment is indicated by the labels (ChF, PF or HPF). Those common for use in all methods do not have these indications.

- Tweezers (DUMONT high precision, Prod #: 5622-NM, 5727, and 5707)
- Dissecting Scope
- Razor blade
- Glass bottom petri dishes (Mattek, P50GC-1.5-14-F Sleeve)
- KCl (Fluka, 60130)
- CaCl_2_ (Merck Millipore, 10043-52-4)
- MgSO_4_ (Merck Millipore, 7487-88-9)
- H_3_BO_3_ (≥99.8%, Roth, 6943.1)
- Ca(NO_3_)_2_ (Merck, 13477-34-4)
- KNO_3_ (Fluka, 60419)
- MES (Roth, 6066.2)
- Merck Millipore: Milli-Q® Integral Water Purification System
- Sucrose for molecular biology
- NaOH (Fluka, 71690)
- Sodium cacodylate trihydrate (Fluka, 20838) ChF CAUTION
- Eppendorf tubes 1.5 and 2 ml
- Glutaraldehyde solution (25%, EM grade, Electron Microscopy Science, 16200) ChF CAUTION
- Osmium tetroxide OsO_4_ (1 g in ampules, Oxkem UK) CAUTION
- Petri dishes
- 1-Hexadecene, aolefin C16 (Tech.), 92%, (ACROS Organics, AC120500010) PF/HPF
- 100 µm thick 6 mm diameter Sapphire disc (Engineering Office of M. Wohlwend, Sennwald, Switzerland) PF/HPF
- Filter paper
- 6 mm diameter aluminium specimen carrier with 150 /150 µm and flat/300 µm recesses (Engineering Office of M. Wohlwend, Sennwald, Switzerland) PF/HPF
- 200 µm thick 6 mm diameter aluminium spacer ring (Engineering Office of M. Wohlwend, Sennwald, Switzerland) PF/HPF
- EM HPM100 high-pressure freezer and specimen cartridge for 6 mm diameter specimen carriers (Leica Microsystems, Vienna, Austria) HPF
- Liquid Nitrogen PF/HPF
- Liquid Ethane PF
- Custom built, gravity driven grid plunge freezer and dedicated tweezers PF
- Forceps (Dumont Switzerland)
- Acetone (HPLC grade ≥99.9%, Sigma, 650501)
- Ethanol (HPLC grade, ≥99.8%, Sigma, 02854)
- Molecular sieves, 3Å beads (Roth, Type 564)
- Leica EM AFS 2 (Leica Microsystems, Vienna, Austria) PF/HPF
- Plastic pipettes
- Epon 812 (Sigma, 45345) CAUTION
- Durcupan ACM (Sigma, 44611) CAUTION
- Dibutylphthalat (Sigma, 80102) CAUTION
- DDSA (2-Dodecenylsuccinic anhydride, Sigma, 45346) CAUTION
- DMP 30 (2,4,6-Tris(dimethylaminomethyl)phenol, Sigma, 45348) CAUTION
- LR white Resin (Agar Scientific, AGR1281) CAUTION
- Teflon embedding moulds with 100 µm depth (custom made)
- Silicon embedding moulds, 14mm(L)x5mm(W)x4mm(D), single tapered end (custom made)
- Toothpicks
- Glass microscope slides, non-coated and coated with Teflon spray
- Teflon (PTFE) spray CAUTION
- Oven 60°C
- Bow saw with 400 nm thick blade
- Araldite® Standard, Turbo® Kleber
- Macroscope (Leica, Z16 APO and Olympus SZ61)
- Glass strips (Leica #16 84 00 31, 400 x 24 x 6.4 mm)
- LKB KnifeMaker 7800 (Ted Pella, Inc, USA)
- Ultra-microtome (Leica Microsystems Reichert Ultracut E and/or Leica Ultracut T)
- Ultra 45° diamond knife (Diatome Switzerland)
- Syringes
- 0.2 µm sterile filter attached
- Slot grids (Agar Scientific, AGG2500C)
- MED 020 (Leica Microsystems)
- TEM grid storage boxes (Ted Pella, Inc, USA, GSB100)
- Kimwipes (Kimtech Science, Kimberly-Clark)
- Formvar 1% (EMS Electron Microscopy Sciences USA, 15830)
- Ethylene chloride (1,2-Dichloroethane ≥99.8%, Sigma, 284505-250ML)
- Parafilm®
- Batzers carbon coater FDU 010
- 1% Toluidine blue
- Double distilled water (ddH_2_O)
- Bright field of phase contrast microscope
- Monoclonal antibodies LM6 and LM15 (Plant Probes, University of Leeds, UK)
- (1-3)-β-D-Glucan antibody (Anti-Callose), Biosupplies Australia Pty Ltd.
- Monoclonal anti-polyHistidine antibody (Sigma, H1029)
- Anti-rat IgG (whole molecule)-gold antibody (Sigma, G7035) and anti-mouse IgG (whole molecule)-gold antibody (Sigma, G7777)
- Uranyl acetate (Fluka, 73943) CAUTION
- Lead(II) nitrate Pb(NO3)2, (Sigma-Aldrich, 228621-100G) CAUTION
- Lead(II) citrate tribasic trihydrate C_12_H_10_O_14_Pb_3_. 3H2O, (Sigma 15326) CAUTION
- Sodium hydroxide pellets NaOH 1N, (Merck, 106482)
- FEI (Philips) CM100 or FEI Tecnai G2 Spirit TEM

## Solutions Setup

### Pollen germination medium

#### Arabidopsis Pollen Germination Medium [A-PGM; prepared per (Boavida & McCormick, 2007)]

Prepare and filter-sterilize stock solutions of 1 M KCl, 1 M CaCl_2_, 1 M H_3_BO_3_, and 1 M MgSO_4_. Stock solutions can be stored at RT for future use. The 1 M H_3_BO_3_ solution will form precipitate over time; to dissolve this, heat the solution in a microwave for few seconds or in a 65°C oven for few minutes and shake thoroughly to dissolve. Before use, prepare 25 ml of fresh A-PGM by adding 125 µl of 1 M KCl, 125 µl of 1 M CaCl_2_, 40.4 µl of 1 M H_3_BO_3_, and 25 µl of 1 M MgSO_4_ to 23 ml of Milli-Q water. Then add 2.5 g of sucrose (MW = 342.3 g/mol) and let it dissolve. Adjust the pH to 7.5 with 0.1 M NaOH (about 30 µl should bring the pH to 7.5) and fill the volume up to 25 ml with Milli-Q water. Aliquots of fresh A-PGM can be stored at -20°C for future use.

### Pollen fixation and embedding media

- **Glutaraldehyde stock solution:** 10 ml of 25% glutaraldehyde EM grade is supplied in an ampule. Create a line of weakness around the neck of the ampule with a glass knife. Then, under the hood, break the head of the ampule away. Make aliquots of 0.5 ml and store at -20°C for future use.
- **Sodium cacodylate stock solution:** Prepare 0.1 M solution by dissolving 133.743 mg of sodium cacodylate trihydrate ((CH_3_)_2_AsO_2_Na · 3H_2_O) in a final volume of 100 ml Milli-Q water. Adjust to pH 7.4 with 1 N HCl. The solution can be stored at 4°C for several weeks. However, check regularly before use that it is still clear and has no precipitates.
- **Fixation working solution (1.25% glutaraldehyde in 0.05 M sodium cacodylate):** Fill half of the final volume with 0.1 M sodium cacodylate buffer. Add 1/20 of the final volume of 25% glutaraldehyde, fill up with Milli-Q water to the final volume. For example: To prepare 10 ml, add 5 ml of 0.1 M sodium cacodylate, 0.5 ml glutaraldehyde 25% and 4.5 ml Milli-Q water, and mix well. Fixation solution should be freshly prepared before use.
- **Osmium tetroxide OsO4 (1% wt/vol):** 0.25 g OsO_4_ crystal is supplied in an ampule. Drop the ampoule in liquid nitrogen to release the crystal from the inner wall. Create a line of weakness around the neck of the ampule with a glass knife. Under the hood, break the head of the ampule away.
- ***For ChF***, dissolve the crystal in 25 ml of Milli-Q water in an Erlenmeyer flask (with ground glass socket and glass stopper). Close the flask and store the solution at 4°C in the dark. The solution can be used for several weeks. If the solution begins to change from colourless clear to black, discard it and prepare a fresh new solution.
- ***For FS***, dissolve the crystal in 25 ml water-free acetone and store in liquid nitrogen.
- **Water-free acetone or water-free ethanol:** Immerse 3Å molecular sieve granules (Roth, Type 564) in pure acetone or ethanol (HPLC grade) and store at RT.

#### **Epon-araldite embedding medium**

*Stock solution:* For 50 ml, weigh 23.63 g of Epon 812, 30.73 mg of durcupan ACM and 2.89 g of dibutylphthalat into a 50 ml Falcon tube and mix by inverting. The stock solution can be stored at RT for several weeks.

*Embedding resin (100%)*: For 10 ml of embedding resin, add 5.57 g of the stock solution, 4.76 g of DDSA and 294 mg of DMP 30 into a 15 ml Falcon tube. Mix the solution by gently inverting. For the final embedding, degas embedding resin in a vacuum degasser for few minutes, and immediately proceed to final embedding.

CRITICAL STEP: The embedding resin starts to polymerize immediately after the addition of the accelerator (DMP 30); it should be used within 60 min.

**PBS**: **1 l of 10x PBS:** add 80 g of NaCl; 2 g of KCl; 17.8 g of Na_2_HPO_4_, 2H_2_O (MW=178 g); 2.4 g of KH_2_PO_4_ into 850 ml of Milli-Q water. Adjust the pH to 7.2 (0.1 N HCl or NaOH) and make up the volume to 1 l with Milli-Q water. Store at RT.

**100 ml of 1x PBS:** add 10 ml of 10x PBS to 90 ml of Milli-Q water, check/adjust the pH to 7.2. Both the 10x and 1x PBS solutions can be stored at RT for several months. Before each use, check that the solution has no precipitates.

**Blocking solution (4% milk in PBS buffer):** For 2 ml of the blocking solution, dissolve 80 mg of milk protein (non-fat milk) in a final volume of 2 ml of PBS. Centrifuge the solution at 10,000 *g* for 1 min and collect the supernatant. Blocking solution should be freshly prepared before use.

**1% Toluidine blue solution (prepared per (Millonig & Millonig, 1976))**: For 40 ml, dissolve 0.4 g Borax (Na_2_B_4_O_7_, 10H_2_O, ≥99.5%, Sigma, B9876) and 0.4 g Toluidine blue O (Chroma, 1B481, Germany) in ddH_2_O and store at RT.

**2% Uranyl acetate**: In a designated hood for handling radioactive substances, prepare 20 ml of 2% uranyl acetate by dissolving 0.4 g in Milli-Q water. Filter to sterilize the solution and store in the dark at 4°C.

**Lead citrate (prepared per (Reynolds, 1963))**: For 50 ml, make the following solutions: dissolve

1. 1.33 g of lead nitrate in 15 ml Milli-Q water,
2. 1.76 g sodium citrate in 15 ml Milli-Q water and
3. 2 pellets (0.4 g) of NaOH in 10 ml Milli-Q water.

In a 50 ml Erlenmeyer flask, add (a) and (b) and shake up thoroughly to form a homogenous milky solution. Add 8 ml of (c) to the flask. The solution should immediately become clear. Fill up the flask to the 50 ml mark with Milli-Q water. Gently mix and store 10 ml aliquots at 4°C.

## PROCEDURE

The experimental setup of the different steps from Arabidopsis pollen germination and fixation to sectioning are schematically represented in Fig. 1 and Fig. 2.

### Pollen germination

#### **Semi-in vivo Arabidopsis pollen germination (TIMING 4 h)**

1. With tweezers, pick flowers that have opened on the same day by the pedicel and, under the dissecting scope, carefully remove the sepals and petals. Pick the filaments of stamens with dehiscent anther and carefully pollinate the stigma. Two tweezers are required here, one to hold the flower at the pedicel and the other to pick the sepals and petals off and for pollination.
2. Cut about 4 pieces of 1cm squares of tissue paper and place them side by side inside of a glass bottom dish (see Fig. 1). Load about 80 µl of Milli-Q water onto the tissue pieces to keep a humid environment inside the dish during pollen germination. Then fill the well of the dish with 200 µl of A-PGM (Fig. 1).
3. Use a new razor blade to cut the pollinated pistil just below the style (about 0.5 mm from stigmatic papillae). Carefully pick it up by the stigma with the tweezers and transfer right away onto the A-PGM in the dish. Ensure that only the base (the ovary end) of the pistil is plunged or in contact with the A-PGM.

TROUBLESHOOTING: The pistil usually sits with the base on the A-PGM when placed at the edge close to the wall of the well and this is okay. Otherwise, use the tweezers to orient the tissue accordingly, while avoiding any damage. This allows for the PTs to grow through the pistil into the A-PGM.

1. After 3.5 h of incubation at 22°C, PTs are seen under the microscope to have grown through into the A-PGM. The emerged tubes are neither too long nor too short after 3.5 h. It is important (except for your specific reasons) not to let the tubes grow too long into the A-PGM as this would increase their tendency to curl around, making it more difficult to straighten them during cryo-fixation and during embedding.

### Pollen tube fixation

#### **Chemical fixation and dehydration**

#### **glutaraldehyde fixation (TIMING 2.5 h)**

Add 250 µl of fresh fixation solution into 2 ml Eppendorf tubes. With tweezers and under the dissecting scope, carefully pick up the specimen from the A-PGM at the stigmatic end and immediately transfer into the fixation solution. Samples are fixed at RT for 15 min and then on ice for 1 h 45 min. PAUSE POINT: Fixation can also be done at 4°C overnight or for days.

TROUBLESHOOTING: The specimen may remain afloat on the fixation solution, which may prevent full contact with and proper penetration of fixative. To get it totally immersed, spin the tube at 10,000 *g* for about 15 secs, then very gently scrape the bottom on an Eppendorf tubes rack to separate any clusters of specimen.

#### **Rinsing**

Rinse specimens with 500 µl Milli-Q water or PBS buffer 3 times for 10 min. Keep the specimen-containing tubes on ice during all the rinsing steps.

#### **Osmium Tetroxide Fixation (TIMING 2 h)**

Replace the washing solution with 250 µl 1% OsO4 and incubate on ice for 2 h. Rinse specimens as described above.

#### **Dehydration with Acetone (TIMING 70 min)**

Still on ice, for 10 min each, perform serial dehydration of the sample in 30% v/v, 50% v/v, 70% v/v, 90% v/v and 2 times in 100% acetone. Then transfer the sample into 100% water-free acetone for another 10 min, still on ice.

#### **Cryo-fixation and freeze substitution**

#### **High pressure freezing (TIMING 15 min)**

1. Place ethanol-cleaned, uncoated, 100 µm thick, 6 mm sapphire disc into the slot of a dedicated middle plate with ridge. Pipette and transfer 10 µl A-PGM onto the disc. Under a dissecting microscope, carefully pick Arabidopsis samples by the stigmatic end with fine tip tweezers and immediately transfer onto the 10 µl PGM.

CRITICAL STEP: Ensure that the PT do not curl around by avoiding the sharp tweezer edge making contacts with the PT. When picked up by the stigma and pulled perpendicular to the surface of the A-PGM, the PT align straight and will remain so even after freeze substitution and embedding.

1. Suck off excess A-PGM using filter paper. Add drops of 1-hexadecene (total volume needed 20–25 µl) to the specimen near the stigmatic end on the Sapphire discs. This ensures that the PTs remain longitudinally aligned and prevents them from curling and sticking to the Sapphire disc.
2. Subsequently, dip the 300 µm recess of the 6 mm diameter aluminium specimen carrier in 1-hexadecene and add on top of the disc with the cavity facing the sample. Complete the sandwich with an additional 200 µm thick 6 mm diameter spacer ring to fully fill the bore of the dedicated middle plate. Immediately freeze the set in an EM HPM100 high-pressure freezer (Leica Microsystems, Vienna, Austria).

PAUSE POINT: Transfer frozen samples into liquid nitrogen and store in an appropriately labelled container until FS.

#### **Plunge freezing (TIMING 15 min)**

Samples were plunge frozen in liquid ethane using a custom built, gravity driven grid plunging device.

1. Pick and hold a 100 µm thick 6 mm diameter Sapphire disc flat with a reverse tweezer dedicated for the grid plunger. Pipette and transfer 10 µl A-PGM on to the disk. Under a dissecting microscope, pick Arabidopsis samples by the stigmatic end and immediately transfer onto the 10 µl A-PGM on the disc.
2. Mount the tweezers onto the arm of the grid plunger and suck away excess medium with filter paper and immediately plunge freeze in liquid ethane held at approximately -180°C (just above melting temperature). Alternatively, add 10 µl of 1-hexadecene to the specimen near the stigmatic end on the Sapphire discs. Suck off excess 1-hexadecene with filter paper. Mount the tweezers with the disc onto the arm of the plunge freezer and immediately plunge freeze.
3. Immediate transfer each frozen sample into liquid nitrogen and store in an appropriate container. We observed no ultrastructural difference between PF specimen with or without 1-hexadecene.

#### **Freeze substitution (TIMING 24 h)**

Using the Leica EM AFS 2, carry out freeze-substitution in water-free acetone with 1% OsO_4_ for 8 h at -90°C, 7 h at -60°C, 5 h at -30°C, 1 h at 0°C, with transition gradients of 30°C per hour. Rinse samples twice with water-free acetone, and proceed to embedding.

### Embedding (TIMING 50–55 h)

1. **Epon infiltration:** Transfer samples into 50/50 Epon embedding resin/water-free acetone and incubate at RT for 2h. Infiltration can also be done overnight at 4°C. Replace the infiltration mixture with 100% Epon embedding resin and proceed to embedding.
2. **LR white infiltration:** Infiltrate samples with LR white in water-free ethanol 33% v/v overnight at 4°C, 66% v/v for 7 h at 4°C, 100% LR white overnight at 4°C and in fresh 100% LR white at RT before polymerization.

**Embedding**

1. Transfer about 150 ml of degassed Epon embedding resin or 75 ml LR white onto the 100 µm deep Teflon embedding mould.
2. Using toothpick or tweezers, carefully transfer the specimen from the 100% Epon onto the mould. Lily specimens would lie flat on the mould. For Arabidopsis, under the dissecting scope, use the tweezers to gently hold and pull the sample in the resin from the stigmatic end by few millimetres to straighten the PTs.
3. Gently, while limiting air bubbles, place a Teflon coated glass slide on the mould over the resin (Fig. 1) and transfer the set into 60–65°C oven for 24 - 48 h.

CRITICAL STEP: The straightening of PTs by this approach is critical in facilitating the subsequent trimming and ultra-sectioning steps: it allows for ultra-thin sections to be cut along the relatively straight PTs for longitudinal view and facilitates the determination of the relative position within the PT for transverse sections. Thus, one can visualize different cytoplasmic and cell wall ultrastructural features across the pollen tube length.

LR white does not polymerise well with air, thus make sure that the specimen is right in the middle of the resin before curing.

1. Fill the cavities of the silicone mould with 100% Epon embedding resin and cure for 48 h at 60 °C, to form empty Epon blocks.

### Preparation of Grids (TIMING 80 min)

1. Formvar-coated grids are commercially available. However, we coat grids ourselves as described. Grids have a shiny and a dark side. Coating with Formvar can be done on any of the sides, but we recommend the smoother shiny side to which Formvar film sticks better.
2. Overfill a staining dish with Milli-Q water and clean the surface by dragging a Kimwipe across the surface.
3. Dip an ethanol cleaned glass slide into 1% Formvar solution two times and immediately suspend in the vapour in a bottle of ethylene chloride solution for about 3 min.

CRITICAL STEP: To achieve a broadly regular thickness of the Formvar film, remove the glass slide from solution at a regular speed. If very fast, the film tends to be too thick and if too slow too thin. An intermediate speed should allow for the adequate film thickness.

1. Score the edges of the slide with a clean razor blade and frost it by breathing onto the slide.
2. Float the thin Formvar film onto the surface of the water by touching the bottom of the slide, held perpendicular to the surface. Under light, regions of different thicknesses of the film are different in colour. Gently lay the grids on the golden regions (which correspond to about 90–120 nm thick).

CRITICAL STEP: The thinner grey regions of the film would break easily during handling while the thicker blue region would reduce the resolution in the TEM.

TROUBLESHOOTING: The Formvar solution, once opened, can be used for several months. However, it may change over time such that the films produced are constantly too thick (blue or green). In this case, replace it with a fresh 1% Formvar solution.

1. Pick up the films and grids with parafilm. Let the grids dry for at least 30 min.
2. Stabilized the film with 8–10 nm thick carbon coat using the MED 020 (Leica Microsystems).

CRITICAL STEP: Check the quality of the grids after carbon coating as the film on some grids may wrinkle. Wrinkled films would eventually introduce wrinkles on ultra-thin sections and cause image focusing difficulties in the TEM.

### Mounting, trimming and ultra-sectioning (TIMING 24 h)

1. Separate polymerized Epon from the mould by gently plugging the slide off (Fig. 2A). Cured Epon sticks to the Teflon-coated slide. Assess the samples under the dissecting microscope to check the alignment of the PTs.
2. Take an image of the sample and measure the length of the PTs from the ovary end to the apex of the longest tubes (Fig. 2B).
3. Cut about 0.5 mm into the empty Epon block on the tapered end using the bow saw.
4. Cut the specimen into a trapezoid shape with a blade and glue with Araldite Standard into the cut on the empty block (with the longer of the parallel sides facing inward) as shown in Fig. 2. The glue hardens overnight.
5. Trim the block into a trapezoid shape around the specimen.

Specimens are cut, glued and trimmed for sectioning following three modes, which define whether transverse or longitudinal sections are produced (Fig. 2C–J). Trimming can be done with an electronic block trimmer or manually with glass knife on the ultramicrotome (the method we used).

TROUBLESHOOTING: Use a new glass knife to trim away excess Epon/Araldite around the specimen on each block. Used glass knife tend to be somewhat blunt and produce rough instead of smooth surfaces and edges, which can make subsequent sectioning quite challenging. LR white resin can get brittle, so trimming and sectioning should be done gently and with care to avoid breaking the block and/or specimen.

Milli-Q water for filling the diamond knife and for subsequent rinsing of grids should be filtered sterilized using a syringe with a 0.2 µm sterile filter attached to avoid contaminating the sections with residual particles.

1. **Sectioning mode 1 (Fig. 2C–E)** Transverse sections are produced from the shank towards the apex of the PTs. Trim the pistil away to section only PTs outside in the resin or allow it to visualize PT within the pistil.
2. **Sectioning mode 2 (Fig. 2F–H)** Transverse sections are produced from the apex towards the shank of the PTs. CRITICAL STEP: Care is required here to avoid having too much Epon/Araldite before the apex of the PTs, but also to avoid trimming the PTs away.
3. **Sectioning mode 3 (Fig. 2I–J)** Longitudinal sections of PTs are produced on flatly glued specimen block.

Tip: Flat embedded samples are about 100-150 µm thick and the PTs are concentrated in the second third region. Thus, trim away the first third (about 30–50 µm) into the block before taking semi-thin sections for toluidine blue staining.

1. Cut semi-thin (200–350 nm thick) sections with the diamond knife and transfer onto glass slides. Stain with a drop of toluidine blue on a 60°C hot plate for about 3 min and wash the stain away. Check under a bright field or phase contrast microscope for PTs (Supp. Fig. 1A–B). If no PT are seen after toluidine blue staining, section about 10 µm into the block and repeat the staining. Repeat this until the tubes are seen on the sections.
2. Then, cut and transfer ultra-thin (60–80 nm thick) sections onto the carbon coated film surface on the slot grids. Record the relative position along the tube during sectioning.

If trimming is done so that a minimal area is left around the specimen, then it is possible to transfer many serial sections onto one grid. Taking this in account, we sometimes could transfer as many as 20 ultra-thin sections onto one slot grid.

### Immunogold labelling (TIMING 5–6 h)

In all the labelling and post-staining steps, drops of solutions are placed on Parafilm.

1. Rehydrate grids with ultra-thin sections by floating the sections-side on drops of PBS blocking solution for 1 h.

CAUTION: For immunogold labelling and subsequent staining of grids with uranyl acetate and lead citrate, take care to avoid breaking the Formvar/carbon coating as this will lead to loss of the sample.

1. Move the grids onto a drop of fresh blocking solution containing an adequate dilution of the primary antibody (1:50 LM6, LM15, 1:150 Anti-callose). PAUSE POINT: Incubate for 2h at RT or overnight at 4 °C.
2. Rinse grids three times in drops of PBS buffer for 10 min to wash away any unbound or loose, unspecifically bound antibodies.

CRITICAL STEP: Make sure that grids do not dry out during any of the incubation steps as this may destroy the primary antibody and obstruct the binding of the secondary gold-coated antibody.

1. Transfer grids onto drops of fresh blocking solution containing 1:25 dilution of 10 nm gold-coated secondary antibody (anti-rat IgG (whole molecule)-gold antibody against LM6, LM15, and anti-mouse IgG (whole molecule)-gold antibody against anti-callose, and incubate for 1 h.
2. Rinse grids 3 times on drops of PBS buffer and 2 times on Milli-Q water for 5 min per wash.

Note. The same resin-embedded sections can also be used for immunogold labelling of other pollen tube epitopes as well as for light/fluorescence microscopy.

### Uranyl Acetate and Lead Citrate staining (TIMING 20 min)

1. Stain grids by floating the section-side on 40 µl drops of 2% uranyl acetate for 10 min.
2. Rinse grids 4 times on drops of Milli-Q water, and dry them by carefully sucking off liquid with a filter paper. Some authors recommend drying grids for several hours after uranyl staining, but our approach of drying on filter papers and proceeding immediately to lead citrate staining produces good contrast.
3. Stain the sections (Supp. Fig. 1C) on drops of lead citrate (prepared per Reynolds, 1963) for 5 min.
4. Rinse 4 times on drops of Milli-Q water and dry the grids by carefully sucking up the liquid with a filter paper
5. Return grids into the grid box and dry at 37°C for 15 minutes.

### TEM imaging (TIMING 1–3 h)

All thin sections were imaged in a CM 100 transmission electron microscope (FEI, Eindhoven, The Netherlands) at an acceleration voltage of 80 kV using a Gatan Orius 1000 CCD camera (Gatan, Munich, Germany).

TROUBLESHOOTING:

1. For immunogold labelled section, it can get confusing at times to distinguish some cellular components like ribosomes that may stain heavily with heavy metals and look like gold particles. This can be resolved by varying the histogram on the live view mode of the camera to make the difference in contrast clearer.
2. Membrane delimitations of membrane-bound structures can be difficult to resolve on one section only due to the dependence on the angle at which the section cuts through the structures. By analysing consecutive serial sections, this problem can be easily resolved as some perspective on the 3D structure can be obtained. Using this approach, we could distinguish lipid droplets from vacuoles in HPF and PF section.
