# Supplementary material for: Efficient preparation of Arabidopsis pollen tubes for ultrastructural analysis using chemical and cryo-fixation
Source: BMC Plant Biol. 2017 Oct 27;17:176. doi: 10.1186/s12870-017-1136-x (PMC5658917; doi:10.1186/s12870-017-1136-x)
Supplement: Supplementary file 2 — Loss of the primary cell wall in PF. PTs fixed by PF frequently showed loss of the primary (outer) cell wall layer, leaving the callose layer. This was not observed with the other fixation methods. Scale bar: 500 nm. (DOCX 250 kb) [file 12870_2017_1136_MOESM2_ESM.docx]

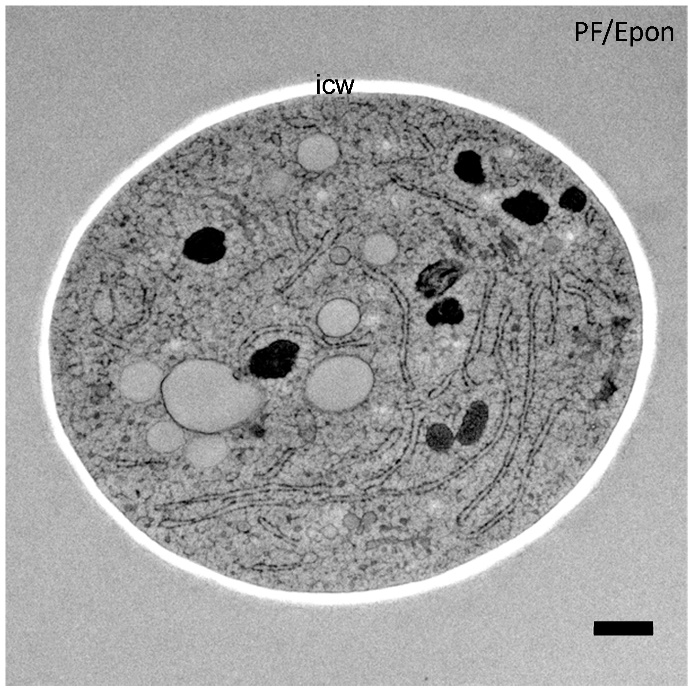


**Additional file 2. Loss of the primary cell wall in PF.** PTs fixed by PF frequently showed loss of the primary (outer) cell wall layer, leaving the callose layer (inner cell wall; icw). This was not observed with the other fixation methods. Scale bar: 500 nm.
