# Supplementary material for: Efficient preparation of Arabidopsis pollen tubes for ultrastructural analysis using chemical and cryo-fixation
Source: BMC Plant Biol. 2017 Oct 27;17:176. doi: 10.1186/s12870-017-1136-x (PMC5658917; doi:10.1186/s12870-017-1136-x)
Supplement: Supplementary file 3 — Comparison of the ChF, PF, and HPF ultrastructural details of PTs within the pistil. The intracellular ultrastructural details of PTs within the TT achieved by HPF (A), PF (B) and ChF (C). Except for the lipid droplets which are best resolved by ChF, the resolution of most intracellular features is highest in HPF, followed by PF and then ChF sections. Ld = Lipid bodies, g = Golgi, er = endoplasmic reticulum, v = vacuole, m = mitochondria Scale bar: 500 nm. (DOCX 531 kb) [file 12870_2017_1136_MOESM3_ESM.docx]

**
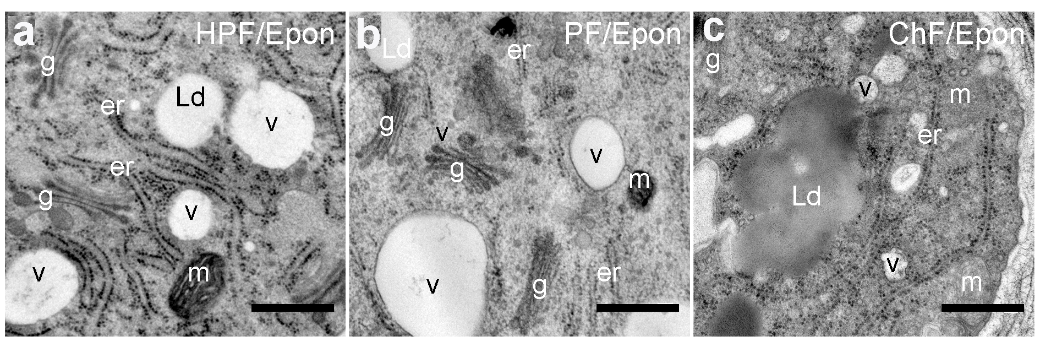
**

**Additional file 3. Comparison of the ChF, PF, and HPF ultrastructural details of PTs within the pistil.** The intracellular ultrastructural details of PTs within the TT achieved by HPF (A), PF (B) and ChF (C). Except for the lipid droplets which are best resolved by ChF, the resolution of most intracellular features is highest in HPF, followed by PF and then ChF sections. Ld = Lipid bodies, g = Golgi, er = endoplasmic reticulum, v = vacuole, m = mitochondria Scale bar: 500 nm.
